# Primary adult sellar SMARCB1/INI1-deficient tumor represents a subtype of atypical teratoid/rhabdoid tumor

**DOI:** 10.1038/s41379-022-01127-2

**Published:** 2022-07-08

**Authors:** Zejun Duan, Kun Yao, Shaomin Yang, Yanming Qu, Ming Ren, Yongli Zhang, Tao Fan, Heqian Zhao, Jie Gao, Jing Feng, Xiaolong Fan, Xueling Qi

**Affiliations:** 1 Department of Pathology, Sanbo Brain Hospital, Capital Medical University, Beijing, 100093 China; 2 Department of Pathology, School of Basic Medical Sciences, Third Hospital, Peking University Health Science Center, Beijing, 100191 China; 3 Department of Neurosurgery, Sanbo Brain Hospital, Capital Medical University, Beijing, 100093 China; 4 Department of Radiology, Sanbo Brain Hospital, Capital Medical University, Beijing, 100093 China; 5 Beijing Key Laboratory of Gene Resource and Molecular Development, Laboratory of Neuroscience and Brain Development, School of Life Sciences, Beijing Normal University, Beijing, 100875 China

**Keywords:** CNS cancer, DNA methylation

## Abstract

Loss of function in *SMARCB1/INI1* has been observed in a group of malignancies collectively defined as SMARCB1/INI1-deficient neoplasms. Primary intracranial SMARCB1/INI1-deficient tumors in adults are extremely rare. We collected eight primary adult sellar SMARCB1/INI1-deficient tumors to study their clinicopathological and (epi)genetic characteristics. We performed a comprehensive assessment of the clinical, radiological, morphological and immunohistochemical features. FISH analysis for the *SMARCB1* locus and target exome sequencing for 425 cancer relevant genes were performed. Furthermore, six bona fide proximal epithelioid sarcoma (PES), fourteen atypical teratoid/rhabdoid tumors (ATRT) in brain and five pediatric poorly differentiated chordomas (PDC) in the clivus were collected for comparative analysis of differential diagnostic maker expression and DNA methylation profile. The median age was 47.1 years, ranging from 26 to 73 years. On morphology, tumors were characterized by sheets of monomorphic larger epithelioid-like cells, in two cases with rhabdoid cells. “Stag-horn” vasculatures were observed in five cases. The loss of INI1 protein expression, co-expression of epithelial makers and mesenchymal markers were observed in all cases. CD34 expression was observed in six cases. Heterozygous deletion of *SMARCB1/INI1* was confirmed using FISH in six cases. The results of target exome sequencing showed three patients harbored heterozygous point mutations in *SMARCB1*. The epigenetic features of the primary adult sellar SMARCB1/INI1-deficient tumors resembled the ATRT-MYC subgroup, but clustered apart from PES and PDC. Based on epigenetic characteristics, primary adult sellar SMARCB1/INI1-deficient tumors represent a subtype of ATRT with similar epigenetic characteristics of ATRT-MYC subgroup. Our findings suggest that DNA methylation profiling should be utilized for differential diagnosis for the majority of epithelioid sarcoma and (sellar) rhabdoid tumor.

## Introduction

The INI1 protein encoded by *SMARCB1* (also known as *INI1*) gene located at 22q11.2 is a central component of the *switch/sucrose*-non-fermentable (SWI/SNF) chromatin remodeling complex, which regulates gene expression important for lineage specification and maintenance of stem cell pluripotency^1,2^. INI1 is ubiquitously expressed within the nuclei of normal cells, where it can be detected by routine immunohistochemistry^3^. SMARCB1 acts as a tumor suppressor gene, and loss-of-function alterations due to various genetic and/or epigenetic abnormalities give rise to SMARCB1-deficient tumors^4^. In 1998, somatic alterations, mainly deletions, in the *SMARCB1* gene accompanying loss of expression were identified as a molecular marker of malignant rhabdoid tumors (MRTs)^5^. Since then, *SMARCB1* gene inactivation or loss of expression has been observed in a group of malignancies collectively defined as SMARCB1/INI1-deficient tumors^2,6^.

Mutations affecting the SWI/SNF complex are documented in 20% of human SMARCB1-deficient neoplasms, which affect patients of a wide age range, from infants to elderly patients in their ninth decades^1,7,8^. Notably, some types of SMARCB1/INI1-deficient tumors can occur intracranially. Atypical teratoid/rhabdoid tumors (ATRTs) are the most common type of SMARCB1/INI1-deficient tumors in the central nervous system (CNS), although these aggressive pediatric brain tumors are very rare. Most ATRTs are located in the brain, rarely at the skull base, such as the sellar region^9^ or clival region^10^.

During the clinicopathological diagnosis, we found eight primary adult SMARCB1/INI1-deficient tumors in the sellar region, and diagnosed them as sellar proximal epithelioid sarcoma (PES) since these tumors fulfilled the following PES criteria: (1) the patients were in their middle age; (2) tumors were located in the sellar region and not involving the brain; (3) morphological features consisted of uniformly atypical epithelial cells and in some cases with rhabdoid cells; (4) the tumors co-expressed mesenchymal marker (Vimentin) and epithelial markers (AE1/AE3 and EMA) accompanied by high CD34 expression; (5) the tumors showed loss of nuclear INI1 protein expression and heterozygous deletions of the *SMARCB1* locus according to FISH analysis. Only one case of PES arising in the base of the skull was reported in literature^11^, but there were several reports of adult sellar region ATRTs^9,12–17^. We found that the eight primary adult sellar SMARCB1/INI1-deficient tumors shared similarities in histomorphology, immunophenotype and genetic abnormalities not only with bona fide PES, but also with the reported adult sellar ATRTs. In this study, our goal was to study the differences and similarities between primary adult sellar SMARCB1/INI1-deficient tumors, bona fide PESs, and ATRTs, and to establish differential diagnosis for primary adult SMARCB1/INI1-deficient tumors in the sellar region. Herein, we summarize the clinicopathologic and (epi)genetic characteristics of eight rare primary adult sellar SMARCB1/INI1-deficient tumors. We also report an in-depth comparison with bona fide PES, ATRT, and PDC.

## Materials and methods

### Tumor samples

Eight patients diagnosed as primary adult sellar SMARCB1/INI1-deficient tumors were collected over an 11-year period (Jan 2008 to Dec 2019) in Sanbo Brain Hospital, Capital Medical University. Three other types of SMARCB1/INI1-deficient tumors, including fourteen ATRT in brain, six extracranial bona fide PES, and five pediatric PDC in the clivus were also collected. Except for the six extracranial bona fide PES from the Third Hospital, Peking University Health Science Center, all ATRT and PDC cases were from Sanbo Brain Hospital. Clinical data were obtained by retrospective examination of medical record. Tumor tissues were fixed in 10% neutral buffered formalin, routinely processed and paraffin-embedded. Sections for genomic analyses and immunohistochemistry (IHC) were prepared from formalin-fixed paraffin-embedded (FFPE) tissue specimens. Haematoxylin and eosin (H&E) stained slides were independently reviewed by two neuropathologists. Approval for conducting this study was obtained from institutional ethics committee at Sanbo Brain Hospital, Capital Medical University.

### Immunohistochemical analysis

Immunohistochemical staining were performed on FFPE sections with the primary antibodies described below and the respective secondary antibodies matching to the primary antibodies. The immunohistochemical staining makers included: (i) typical epithelioid sarcoma (ES) markers: AE1/AE3 (Dako, 1:100 dilution), epithelial membrane antigen (EMA) (Dako, 1:250 dilution), Vimentin (Abcam, 1:150 dilution), CD34 (Dako, 1:100 dilution), INI1 protein (Santa Cruz Biotechnology, 1:500 dilution)^18^; (ii) markers for tumors of neural origin: glial fibrillary acidic protein (GFAP) (Dako, 1:400 dilution), OLIG2 (Dako, 1:250 dilution), S-100 (Dako, 1:100 dilution), MAP2 (Abcam, 1:100 dilution), synaptophysin (Syn) (Abcam, 1:200 dilution), chromogranin-A (CgA) (Dako, 1:100 dilution), CD56 (Dako, 1:200 dilution), NeuN (Zeta, 1:100 dilution), Neurofilament (NF) (Covance Research Products, 1:200 dilution); (iii) markers for myogenic tumors: SMA (Cell Signal Technology, 1:150 dilution), Desmin (Ventana, 1:150 dilution), Myoglobin (Leica, 1:250 dilution), MyoD1 (Cell marque, 1:150 dilution), Myogenin (Leica, 1:200 dilution), Actin (Cell Signal Technology, 1:200 dilution); (iv) STAT6 (Abcam, 1:500 dilution) for hemangiopericytoma (HPC), HMB45 (Dako, 1:30 dilution) and MelanA (Novocastra, 1:50 dilution) for melanoma, and CD30 (Dako, 1:200 dilution) for anaplastic large cell lymphoma (ALCL); (v) TP53 (Santa Cruz, 1:200 dilution) for prediction of P53 mutation, and Ki-67 (MIB-1, Labvision, 1:50 dilution) for evaluation of the tumor proliferation activity; (vi) pituitary hormones including GH, PRL, TSH, ACTH, FSH and LH (Ventana, 1:50 to 1:3000 dilution); (vii) other markers for distinguishing primary adult sellar SMARCB1/INI1-deficient tumors, bona fide PES, ATRT, and PDC: ERG (Origene, 1:200 dilution), SALL4 (Biocare, 1:1000 dilution), β-Catenin (Origene, 1:200 dilution), FLI-1(Zeta, 1:50 dilution), brachyury (abcam, 1:5000 dilution); (viii) c-myc (abcam, 1:500 dilution) for evaluating the expression of c-myc protein. The extent of immunoreactivity was graded according to the percentage of positive tumor cell and whether the positive staining was located in the nucleus, plasma or membrane (0: no staining; 1+: <5%; 2+: 5–25%; 3+: 26–50%; 4+: 51–100%)^19,20^.

### Fluorescence in situ hybridization (FISH) analysis

FISH analysis was performed on 4 μm FFPE sections using a laboratory-developed dual-color probes with SMARCB1 as the target (located at 22q11, red fluorochrome, from Anbipin, Guangzhou, China) and EWSR1 as the control (located at 22q12, green fluorochrome, also from Anbipin, Guangzhou, China). The sections were pre-treated in 60 °C oven for 30 minutes, deparaffinized in xylene/isopropanol, and pressure cooked in citrate buffer for 60 minutes. Following digestion in a coplin jar with 4 mg/ml pepsin at 37 °C for 5–10 minutes, the sections were hybridized with the denatured probes at 37 °C for 20 hours. Then the slides were washed and counterstained with DAPI. Hybridization signals were analyzed using an Olympus fluorescence microscope equipped with applied imaging software. A cut-off value for heterozygous deletion of SMARCB1 was set at 30% of the cells with 1 signal for SMARCB1 probe and 2 signals for the control probe. Two experienced pathologists independently reviewed the FISH images and interpreted the results.

### Targeted exome sequencing

Targeted exome sequencing was performed for four cases (cases 3, 5, 6 and 7) with sufficient sample materials according to previously described protocol^21^. FFPE samples were used for DNA extraction using the QIAamp DNA FFPE Tissue Kit (Qiagen). Hybridization based target enrichment was carried out with GeneseeqOne™ pan-cancer gene panel (425-cancer-relevant genes including *SMARCB1*, *SMARCA4*, and *EWSR1* genes, Geneseeq Technology Inc.) (Supplementary Table 1), and xGen lockdown hybridization and washing reagents kit (Integrated DNA Technologies). The libraries were sequenced on the HiSeq4000 platform (Illumina, San Diego, CA) with 2 × 150 bp pair-end reads. Sequencing data were demultiplexed by bcl2fastq (v2.19), analyzed by Trimmomatic^22^. The genome analysis toolkit (GATK)^23^ was used to perform local realignments around indels and base quality reassurance. SNPs and indels were called using VarScan2^24^ and haplotype caller UnifiedGenotyper in GATK, with the mutant allele frequency (MAF) cutoff as 0.5% for tissue samples and a minimum of three unique mutant reads. Common variants were removed using dbSNP and the 1000 Genome project.

### DNA methylation profiling

In order to explore the DNA methylation profiles of the primary adult sellar SMARCB1/INI1-deficient tumors, DNA methylation was assessed using the Illumina Infinium Human Methylation 850 (850k) BeadChip (Illumina, San Diego, USA) at Single Cell Biotech Co. Ltd. (Beijing, China). The cohort included eight primary adult sellar SMARCB1/INI1-deficient tumors, six bona fide extracranial PES, fourteen ATRT, and five PDC samples. First, DNA was extracted from FFPE samples using ReliaPrep FFPE gDNA Miniprep Kit (Promega, WI, Germany), and then restored using Illumina HD FFPE Restoration Kit (Illumina, CA, USA) according to manufacturer’s instructions. Second, DNA underwent bisulfite conversion, amplification, fragmentation, and hybridization to the 850 K BeadChip (Illumina, CA, USA) following the manufacturer’s protocols. Third, raw data were generated using iScan array scanner and preprocessed using GenomeStudio software. Samples with a detection ratio of CpG sites (detection *p* value < 0.05) greater than 95% were used for subsequent analysis. Six samples (1 primary adult SMARCB1/INI1-deficient tumor, 3 bona fide extracranial PES, and 2 PDC) were excluded because of the detection ratio of CpG sites was lower than 95%. An outlier (ATRT_5) was removed. Data preprocessing and normalization were performed using the minfi package, beta and M values were calculated. Probes with the following features were excluded from further analysis: (i) probes with a detection *P* value > 0.01 (the detection p-values were calculated as the total signal (M + U) for each probe to the background signal level, which was estimated from the negative control probes.), (ii) probes for genes located on X and Y chromosomes, (iii) probes containing SNPs (SNP 147), and (iv) probes mapping to multiple genome locations. Finally, 641,483 probes were kept for analysis. Unsupervised hierarchical clustering was performed using the 5000 most variably methylated probes as described by Johann et al.^17^. Unsupervised hierarchical cluster and t-stochastic neighbor embedding (t-SNE) were performed with the hclust and Rtsne package in R 4.0, respectively.

The CNS tumor dataset^25^ (including 91 entities in the training set of over 2800 neuropathological tumors, GSE90496) and sarcoma dataset^26^ (including 62 types of soft tissues and bone sarcomas in the training dataset of 1077 methylation profiles) were used as external datasets to assess the similarities with other tumors using t-SNE analysis (Rtsne, R package,v.0.15). The parameters were set as previously described.

### Statistical significance analysis

Data were analyzed using SPSS Statistics 24.0 software. Statistical significance was examined using Fisher’s exact test.

## Results

### Clinical findings of adult sellar SMARCB1/INI1-deficient tumors

**Fig. 1:**
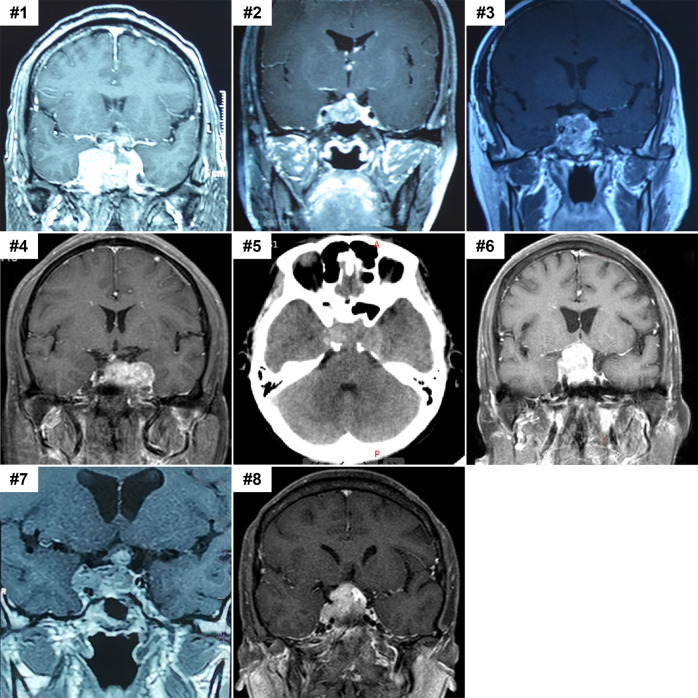
Pre-operative imaging features of the eight primary adult sellar SMARCB1/INI1-deficient tumors. Images shown are pre-operative magnetic resonance images (cases #1–4 and #6–8) and CT image (case #5). All tumors were lobulated or lumpy solid masses located in the sellar region involving intrasella, suprasella or parasella. After enhancement, the lesions displayed homogeneous or heterogeneous contrast enhancement in cases #1–4 and #6–8, CT image showed slightly hyperdense signals in case #5.

In this cohort, we collected eight primary adult sellar SMARCB1/INI1-deficient tumors from six female and two male patients^9,17^, their ages ranged from 26 to 73 (Table 1). Symptoms mainly included headaches, accompanied by ptosis (five cases), visual impairment (fours cases) and nausea and vomiting (three cases). One patient suffered from polydipsia and another one had facial numbness. Endocrine disturbance was observed in 7 of 8 patients with decreased thyroid hormones and TSH. Decreased levels of serum insulin growth factor-1 were found in two cases, and decreased serum cortical hormone only in one case. Radiological images showed the tumors as lobulated or lumpy solid masses involving the intrasellar, suprasellar and parasellar region (cavernous sinus and the third ventricle) (Fig. 1). Involvement of cavernous sinus was observed in seven cases. Tumor protruded into the third ventricle in three cases and involved in the wall of third ventricle in one case. On computed tomography (CT), the tumors were variably isodense or slightly hyperdense. Among them, bone destruction at the base of sella turcica and slope was observed in case 5. On T1 and T2-weighted imaging, the tumors presented as hypointense or hyperintense masses. After enhancement, all masses displayed homogeneous or heterogeneous contrast enhancement. Clinical presentation of patients appeared to be dependent on the location and the size of tumors.

**Table 1:** Clinical features of the eight primary adult sellar SMARCB1/INI1-deficient tumors.

Case #	Sex	Age (y)	Tumor location	Symptoms	Surgery and extent of resection	Treatment (radiation and chemotherapy)	Follow-up and overall survival outcome
1	F	47	Sellar region, bilateral cavernous sinus and the front of the third ventricle	Headache, nausea and vomiting, polydipsia, ptosis and diplopia	Craniotomy, gross total	None	About 1 month; deceased
2	F	52	Sellar region, bilateral cavernous sinus and the front of the third ventricle	Headache, ptosis and fever	First surgery: endoscopy; Second surgery: craniotomy, subtotal	None	2.5 months; deceased
3	M	47	Sellar region, right cavernous sinus	Headache, ptosis, blindness, diplopia, nausea and vomiting.	First surgery: endoscopy; Second surgery: craniotomy, subtotal	Gamma knife after first surgery, NA after second surgery	6 months; deceased
4	F	46	Sellar region, left cavernous sinus	Headache, ptosis, nausea and vomiting.	Craniotomy, gross total	NA	6 months; deceased
5	F	45	Sellar region, bilateral cavernous sinus	Headache, ptosis, diplopia and facial numbness.	Endoscopy, subtotal	None	1 month; deceased
6	F	73	Sellar region, right cavernous sinus	Headache, and decreased vision.	Endoscopy, gross total	NA	3 months; deceased
7	M	26	Sellar region, right cavernous sinus and the wall of third ventricle	Headache and fever	First surgery: endoscopy; Second surgery: craniotomy, subtotal	None	3 months; deceased
8	F	41	Sellar region	Headache	Craniotomy, gross total	Radiotherapy	12 months; alive; cerebellar metastasis

*NA* not available.

Surgical resection by endoscopy or craniotomy was performed in all eight patients. The tumors were grossly resected. No cerebrospinal dissemination and extracranial metastases were observed at the time of diagnosis in any of the eight patients. These cases were followed up for 1 to 12 months (Table 1). Seven patients died within 6 months after surgery, only one survived longer than 12 months even with cerebellar metastasis.

### Histological findings

**Fig. 2:**
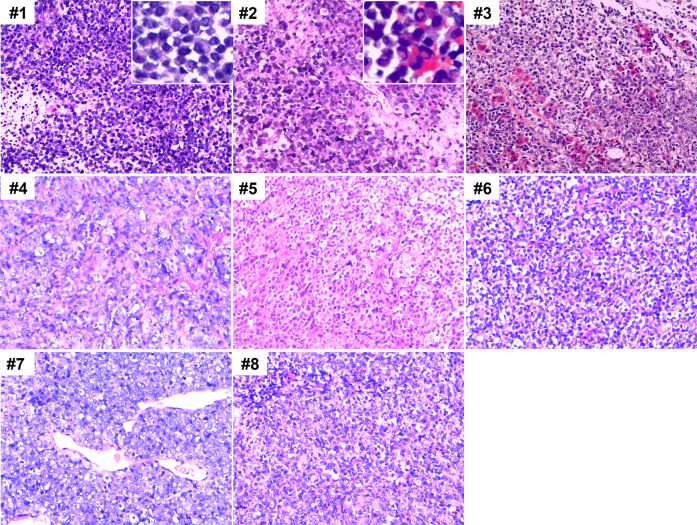
Histologic features of the eight primary adult sellar SMARCB1/INI1-deficient tumors. Images of representative HE staining at a magnification of 200 x are shown. All samples showed high proliferation of atypical epithelial cells with large vesicular nuclei, one or more prominent centrally located nucleoli, and abundant cytoplasm. Rhabdoid cells were seen in cases #1 and #2. Tumor involvement in pituitary gland was found in case #3 (white arrow indicating residual glandular pituitary). HPC-like stag-horn vasculatures were observed in case #7.

The tumors consisted of monomorphic atypical epithelial cells, occasionally accompanied by rhabdoid cells, which were different from ATRT containing variable components with primitive neuroectodermal, mesenchymal, and epithelial features (Fig. 2). The atypical epithelial cells were characterized by large vesicular nuclei, one or more prominent centrally located nucleoli, and abundant cytoplasm. Conspicuous mitotic activity and necrosis were detected in all cases. Hemangiopericytoma-like (HPC-like) stag-horn vasculatures were observed in five cases. Tumor involvement of the pituitary gland was found in four cases. Rhabdoid cells with eccentrically placed nuclei, abundant eosinophilic cytoplasm, and intracytoplasmic, paranuclear hyaline inclusions were observed in two cases. Tumor cells under the nasal sinus mucosa were detected in one case.

### A broad spectrum of immunohistochemical reactivies in primary adult sellar SMARCB1/INI1-deficient tumors

The staining results of diagnosis-related markers showed a broad spectrum of immunohistochemical reactivities (Table 2 and Supplementary Fig. 1). The tumors exhibited typical epithelioid sarcoma immunohistochemical features. EMA staining and diffusely strong staining of Vimentin were observed in all cases. AE1/AE3 and CD34 reactivity were identified in 87.5% (7/8) and 75% (6/8) of tumors, respectively. The tumor cells were characteristically negative for SMARCB1/INI1 expression, which was however positive in vascular endothelial cells as an internal staining control. S-100, GFAP, and OLIG2 were consistently negative in all cases. Other neural markers showed sporadic expression pattern. Syn expression was positive in seven cases, six of which were diffusely positive and one in approximately 40–50% of the tumor cells. MAP2 was diffusely positive in two cases. CD56 expression was found in 10–50% of cells in three cases. Different myogenic markers were positive in five cases. About 10% to 70% of tumor cells expressed Actin or SMA in three cases. Desmin staining was observed only in about 60% of the tumor cells in case 6. Myoglobin, MyoD1, and Myogenin were negative in all cases. The Ki-67 index was uniformly high, ranging from 30% to 70%. TP53 was strongly expressed in more than 50% tumor cells in all cases. C-myc was strongly expressed in five of six cases, one case showed staining in less than 5% tumor cells, the remaining two cases were not stained because no sufficient materials were available. Reticulocyte staining showed abundant reticular fibers in all cases. The pituitary hormones, STAT6, HMB45, MelanA, and CD30 were negative in all cases.

**Table 2:** Histological, immunohistochemical and genetic features of eight primary adult sellar SMARCB1/INI1-deficient tumors.

Case #	Histopathology		Immunohistochemistry								FISH for SMARCB1 loss	Targeted exome sequencing for SMARCB1
	Rhabdoid cells	Hemangiopericytoma-like vasculatures	AE1/AE3	EMA	Vimentin	CD34	INI1 protein	Ki67	Neural markers (GFAP, OLIG2, S-100, NeuN, NF, MAP2, CgA, Syn, CD56)	Myogenic markers (Actin, SMA, Desmin, MyoD1, Myogenin)		
1	Yes	Yes	4+	4+	4+	2+	0	50%	Syn 3+	0	+	NP
2	Yes	No	2+	2+	4+	2+	0	50%	Syn 4+, CD56 2+	Actin 2+	+	NP
3	No	Yes	3+	3+	4+	4+	0	70%	Syn 4+, CD56 3+	SMA 2+	+	Mutation c.544 C > T(p.Q182*)
4	No	Yes	2+	2+	4+	0	0	50%	0	0	-	NP
5	No	No	3+	2+	4+	0	0	50%	Syn 4+, MAP2 4+	SMA 4+, Actin 4+	+	Mutation c.110 G > T(p.R37L) and c.109del(p.R37Vfs*18)
6	No	No	0	3+	4+	4+	0	50%	Syn 4+, MAP2 4+	Desmin 4+	+	No mutation#
7	No	Yes	4+	2+	4+	2+	0	60%	Syn 4+	Actin 2+	-	Mutation c.152 G > A(p.W51*)
8	No	Yes	2+	2+	4+	4+	0	30%	Syn 4+, CD56 3+	0	+	NP

The extent of immunoreactivity was graded according to the percentage of positive tumor cell (0, no staining; 1+, <5%; 2+, 5–25%; 3+, 26–50%; and 4+, 51–100%). -: negative, +: positive. *NP* not performed. # with deletion of *SMARCB1* (copy number: 0.2472).

### Differential CD34 expression between primary adult sellar SMARCB1/INI1-deficient tumors and ATRT

To identify diagnostic markers distinguishing between primary adult sellar SMARCB1/INI1-deficient tumors and other types of SMARCB1/INI1-deficient tumors, we also collected six bona fide PES, 14 ATRT in brain, and five pediatric PDC (Supplementary Table 2). Previous studies proposed a number of markers distinguishing MRTs (including ATRT) from PESs, including CD34, SALL4, ERG, FLI-1, β-catenin, glypican-3, and D2-40^19,20,27,28^. In addition, brachyury expression can differentiate PDC from ATRT^29,30^. We chose frequently used CD34, SALL4, ERG, FLI-1, β-catenin, and brachyury as makers to differentiate the four types of SMARCB1/INI1-deficient tumors in our cohort. The results of immunohistochemical staining are summarized in Supplementary Table 3.

**Fig. 3:**
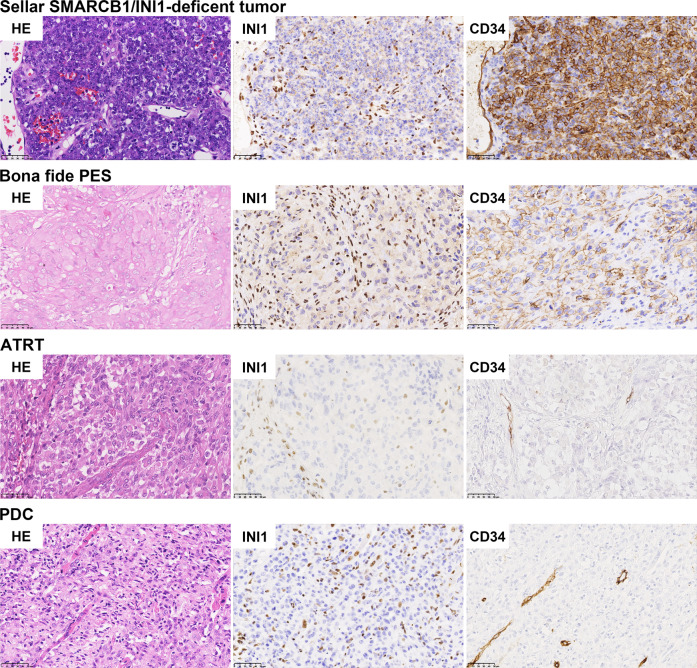
CD34 can serve as a differential diagnostic marker for primary adult sellar SMARCB1/INI1-deficient tumors. All four types of tumors harbor loss of SMARCB1/INI1 expression. Diffuse and strong membrane staining of CD34 was observed in six sellar SMARCB1/INI1-deficient tumors and all bona fide PES. Except for three ATRT and one PDC showed faint and focal CD34 positive expression, CD34 was negative in most ATRT and PDC samples examined.

CD34 staining was strong and diffuse in six out of the eight primary adult sellar SMARCB1/INI1-deficient tumors examined. CD34 staining was also positive in all six bona fide PES. The ratio of CD34 expression was significantly higher in primary adult sellar SMARCB1/INI1-deficient tumors (75%) than that in ATRT (21.4%) (*p* < 0.05, Fisher’s exact test) (Fig. 3). CD34 staining was faint and focal in three ATRTs and one PDC. In contrast, brachyury staining was positive only in PDC samples, and was negative in other three SMARCB1/INI1-deficient tumor types. No distinct staining patterns were observed for SALL4, ERG, FLI-1 and β-catenin. Our findings suggest that CD34 can be used as an effective marker for differential diagnosis between primary adult sellar SMARCB1/INI1-deficient tumors, ATRT in brain, and pediatric PDC. Unfortunately, we could not define whether the expression of CD34 was more frequently encountered in adult ATRTs compared with pediatric ATRTs due to limited number of samples. Brachyury could serve as a reliable marker distinguishing pediatric PDC from other three SMARCB1/INI1-deficient tumor types.

### Deletions and mutations of SMARCB1 in primary adult sellar SMARCB1/INI1-deficient tumors

Heterozygous deletion of *SMARCB1* was detected in six cases by FISH analysis (Supplementary Fig. 2, Table 2). Notably, although *SMARCB1* deletion was not detected in the remaining two cases (cases 4 and 7), SMARCB1/INI1 protein was not detected by IHC in all these samples. In four cases, targeted exome sequencing of 425 cancer related genes was performed for the detection of single nucleotide variations and copy number variations (CNVs, Table 2). In addition to the loss of one allele as shown in FISH analysis, cases 3, 5 also harbored point mutations (c.544 C > T (p.Q182*) [exon 5], c.110 G > T(p.R37L) and c.109del (p.R37Vfs*18) [exon 2], respectively). Case 7 harbored heterozygous point mutation at c.152 G > A(p.W51*) in exon 2.

Other alterations were also detected in several cases, including *GNAS* mutation and *MCL1* amplification in case 3, mutations in *BRCA2* and *NF1* in case 5, mutations in *ATRX*, *EGFR*, *GATA3* and *PTPN13* in case 6. In addition, although high TP53 and c-myc immunohistochemical stainings were observed in almost all cases, mutations in *TP53* and *C-MYC* were not detected.

### Primary adult sellar SMARCB1/INI1-deficient tumors clustered into the pediatric ATRT-MYC subgroup

**Fig. 4:**
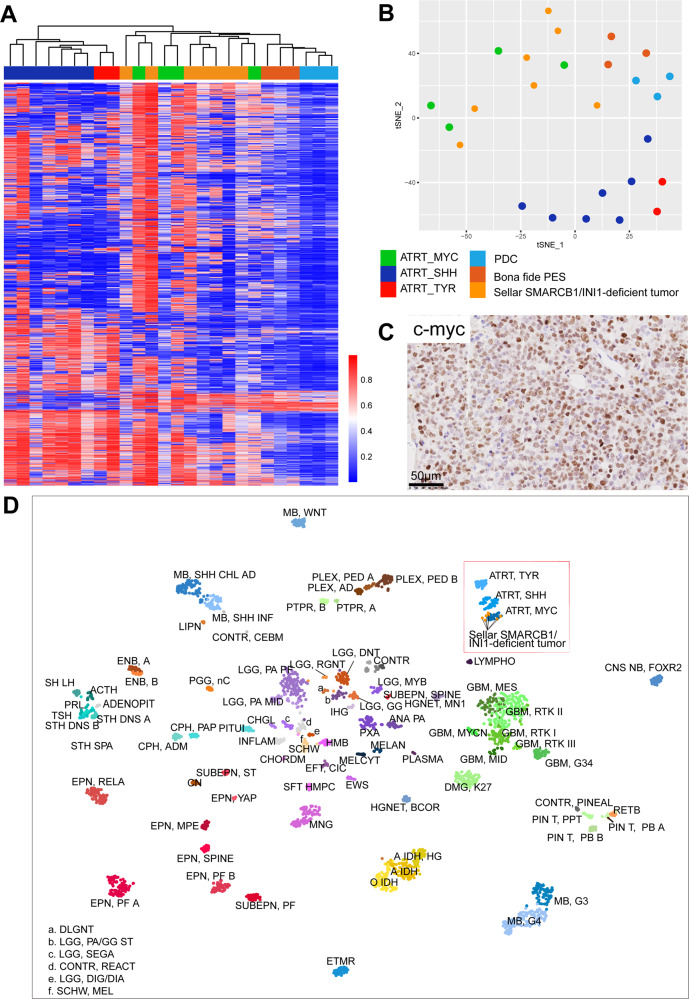
Epigenetic profiling of primary adult sellar SMARCB1/INI1-deficient tumors. **A** Heatmap of unsupervised hierarchical clustering and **B** t-SNE plot of DNA methylation profiles of seven primary adult sellar SMARCB1/INI1-deficient tumors, three bona fide extracranial PES, fourteen ATRT and three pediatric PDC using the 5000 most differentially methylated probes. Seven primary adult sellar SMARCB1/INI1-deficient tumors clustered together with ATRT-MYC subgroup. **C** Diffuse positive c-myc protein staining in primary adult sellar SMARCB1/INI1-deficient tumors. **D** t-SNE cluster of DKFZ/Heidelberg CNS tumor dataset^25^ and our primary adult sellar SMARCB1/INI1-deficient tumors based on the DNA methylation data.

To investigate the resemblance or distinction between primary adult sellar SMARCB1/INI1-deficient tumors and other SMARCB1/INI1-deficient tumors, we generated methylome profiles for seven primary adult sellar SMARCB1/INI1-deficient tumors, thirteen ATRT in brain, three bona fide PES, and three pediatric PDC. Based on the 5000 most variably methylated probes, we first performed unsupervised hierarchical clustering and t-SNE analyses. The results showed that primary adult sellar SMARCB1/INI1-deficient tumors were clustered with ATRT, particularly with the ATRT-MYC subgroup, while pediatric PDC and three bona fide PES were grouped separately (Fig. 4A, B). Pediatric ATRT-MYC subgroup showed elevated MYC expression^31^. In our cohort, 5 of the 6 cases had high expression of c-myc protein (Fig. 4C) as assessed using IHC, though no copy number variations were detected in *MYC* gene by copy-number variation analysis from the methylation array data.

Using the DKFZ/Heidelberg CNS Tumour Classifier^25^ (v11b4), the methylation subtype of the adult sellar SMARCB1/INI1-deficient tumors in our cohort was also determined. In concordance with the findings of unsupervised hierarchical clustering and t-SNE analyses, a high correlation between adult sellar SMARCB1/INI1-deficient tumors and ATRT-MYC tumors was also found (Fig. 4D). Thus, adult sellar region SMARCB1/INI1-deficient tumors have similar methylation profiles with the pediatric ATRT-MYC subgroup.

Using methylation-based classifier for soft tissues and sarcoma^26^, the degree of similarity between primary adult sellar SMARCB1/INI1-deficient tumors and other primary tumors, including 91 brain tumors and 62 sarcomas, was also assessed. Besides pediatric ATRT, PDC and bona fide PES described above, it is well known that SMARCB1/INI1-deficiency occurs in a variety of tumors, including malignant rhabdoid tumor (MRT), epithelioid sarcoma (ES), malignant peripheral nerve sheath tumor (MPNST), extraskeletal myxoid chondrosarcoma (EMCS). Except MRT (occurring in CNS known as ATRT^32^), no significant correlation between primary adult sellar SMARCB1/INI1-deficient tumors and sarcomas was observed (Supplementary Fig. 3).

In summary, primary adult sellar SMARCB1/INI1-deficient tumors have similar DNA methylation profiles with the pediatric ATRT-MYC subgroup, however, primary adult sellar SMARCB1/INI1-deficient tumors are different from bona fide (P)ES, PDC, and other CNS brain entities and sarcomas.

## Discussion

Here, we report eight primary adult sellar SMARCB1/INI1-deficient tumors initially diagnosed as sellar PES. We found that the eight sellar SMARCB1/INI1-deficient tumors shared similarities in morphology, immunohistochemical features and genetic alteration (*SMARCB1* gene) with bona fide PES and reported adult sellar ATRT. Among the eight cases, five cases harbored HPC-like stag-horn vasculature. Six cases were positive for CD34 expression. Furthermore, six cases had heterozygous deletions of the *SMARCB1* locus according to FISH analysis. *SMARCB1* mutations were observed in all three of four cases sequenced. According to epigenetic analysis, primary adult sellar SMARCB1/INI1-deficient tumors showed epigenetic characteristics similar to ATRT-MYC subgroup^17^, but clustered apart from bona fide PES. Overall, primary adult sellar SMARCB1/INI1-deficient tumors should be considered a subtype of ATRT, but not PES on the basis of epigenetic analysis, which is a powerful approach for CNS tumor classification^17^. In addition to the reported features of adult sellar ATRTs, high CD34 expression is also a potential marker for differential diagnosis.

Given the rarity of primary adult sellar SMARCB1/INI1-deficient tumors, the diagnosis was difficult and it was challenging to classify the subtype of SMARCB1/INI1-deficient tumors. On the basis of the morphological and immunophenotype characteristics, we re-considered the diagnosis of several previously reported types of intracranial SMARCB1/INI1-deficient tumors, such as PES^11,33^, adult ATRT^9^, and PDC^34^. PDC is a special entity of chordoma which often affects children under 5 years of age and occurs in the clivus region with notochordal marker brachyury nuclear positive staining^29,34^. Primary adult sellar SMARCB1/INI1-deficient tumors can be discriminated from cribriform neuroepithelial tumor (CRINET) and PDC, but it has been difficult to ascertain whether primary adult sellar SMARCB1/INI1-deficient tumors are ATRT or PES. In addition to ATRT, PES may also be encountered in the base of skull^11^.

### Comparison with bona fide PES

In practice, differentiating primary adult sellar SMARCB1/INI1-deficient tumors from PES can be problematic because they share similar features of morphology, immunohistochemistry and genetic alteration.

We consider the following four aspect leading to the diagnosis of primary adult sellar SMARCB1/INI1-deficient tumors as PES. (1) PES was proposed by Guillou et al.^35^, which are rare, undifferentiated soft-tissue sarcomas, arising in the deep soft tissue^36,37^. PES mainly affects the middle-aged and elderly patients^36,37^. (2) Morphologically, PES is characterized by sheets of large, polygonal cells with variable rhabdoid morphology^36,37^. In the case series described in this paper, primary adult sellar SMARCB1/INI1-deficient tumors had similar morphological features with PES, consisting of uniformly atypical epithelial cells and occasionally with rhabdoid cells. However, we found that five of the eight cases harbored HPC-like stag-horn vasculature. (3) On IHC staining, the most prominent characteristic of PES was co-expression of mesenchymal marker (Vimentin) and epithelial markers (AE1/AE3 and EMA)^36,37^, often accompanied by CD34 positive expression (about 45–50%)^36,37^. In our cohort, up to 75% of cases showed the expression of CD34. (4) Approximately 90% of PES exhibited a loss of nuclear SMARCB1 immunopositivity due to alterations of the *SMARCB1/INI1* gene. At the DNA level, *SMARCB1/INI1* alterations in PES show miscellaneous genetic abnormalities involving mutations or deletions at chromosome 22q^36,37^, but the frequency is significantly lower than that in ARTR^36^. Complete loss of SMARCB1 protein expression was observed in all eight cases in this study. Six cases had a heterozygous deletion of the *SMARCB1* locus according to FISH. DNA sequencing indicated compound heterozygous point mutations at the *SMARCB1* locus in three of the four cases sequenced.

Accordingly, we initially diagnosed the eight primary adult sellar SMARCB1/INI1-deficient tumors as PES based on their occurrence in middle-aged and elderly people, loss of expression of INI1 protein, high expression of CD34, and their location in the sellar region.

### Comparison with adult seller ATRT

ATRTs are rare highly malignant CNS embryonal tumors, usually found in infants and young children, especially those younger than 3 years old, with poor prognosis^38^. ATRTs have been rarely reported in adults. They occur in both supratentorial and infratentorial regions of the brain. Most tumors contain variable components with primitive neuroectodermal, mesenchymal, and epithelial features^38^. The genetic hallmarks of ATRTs are associated with deletion or mutation of *SMARCB1/INI1*^31,38^. Johann et al.^31^ classified ATRTs into three distinct epigenetic subgroups, termed ATRT-TYR, ATRT-SHH, and ATRT-MYC. The sellar or suprasellar region is the second most preferred site for adult ATRT following the cerebral hemispheres^9,39^. About 21 cases of adult seller ATRT have been reported to date^9,12,14,17,31,39,40^. In contrast to their pediatric counterparts, adult sellar ATRTs form a clinically distinct entity with specific clinicopathological, morphological and genetic characteristics, but they nevertheless share a similar DNA methylation profile with the pediatric ATRT-MYC subgroup^17^.

Adult sellar ATRTs have several features different from classical pediatric ATRT. (1) In terms of the sex distribution, sellar ATRT exclusively occurs in adult (20 to 69 years old) with a distinct female preponderance^9,16,39^ and this has never been described for pediatric cases^39^. (2) On histology, different from classical pediatric ATRT with divergent differentiation along neuroectodermal, mesenchymal, and epithelial lineages, most adult sellar ATRTs are composed of relatively uniform population of medium to small-sized tumor cells frequently with rhabdoid cells^9,15,39^. HPC-like stag-horn vasculature seems to be a characteristic structure that is uncommon in pediatric ATRT^9,31^. (3) Neoplastic cells also provide immunohistochemical evidence of divergent differentiation along neuroectodermal, mesenchymal, and epithelial lineages^38^. In the sellar ATRTs, high-specific neuronal (synaptophysin, chromogranin) and glial markers (GFAP) were almost negative^9,13,14^. Although some cases showed expression of S-100^39^, it was negative in most cases^13,14^. The number of CD34 expression positive cases was small, accounting for only 27% in adult ATRT^16^. Unfortunately, the positive expression of CD34 in sellar ATRT was mentioned in only one case^14^. (4) The biallelic *SMARCB1/INI1* alterations, such as homozygous deletions, one allele mutation with the loss of the second allele, and different mutations in each allele, are the genetic hallmark of ATRT^38,41^. The main *SMARCB1/INI1* alterations in sellar ATRT were compound heterozygous mutations and splice-site mutations, but some cases showed no aberrations^9,33,40^. Although several open questions about sellar adult ATRT remain to be clarified, adult ATRT in sellar region may represent a clinicopathologically and genetically distinct variant of ATRT showing a characteristic morphology, different patterns of SMARCB1 alterations, and a histological feature with a unique vasculature^9^.

A comparison of primary adult sellar SMARCB1/INI1-deficient tumors with adult sellar ATRT revealed many clinicopathological and genetic similarities between them, except individual sellar ATRT with GFAP expression and *SMARCB1/INI1* biallelic alterations^9^. Additionally, there was high expression of CD34 in our collection of adult sellar SMARCB1/INI1-deficient tumors.

### Diagnosis based on epigenetic profiles

To verify the diagnosis of primary adult sellar SMARCB1/INI1-deficient tumors, we carried out DNA methylation analysis in seven cases together with samples of fourteen ATRT, three extracranial bona fide PES, and three pediatric PDC. In unsupervised hierarchical clustering analysis of DNA methylation profiles, the seven sellar SMARCB1/INI1-deficient tumors clustered with ATRT-MYC subgroup as reported by Johann et al.^36^, but clustered apart from bona fide PES and pediatric PDC. Moreover, they had an elevated c-myc protein expression which was reported in pediatric ATRT-MYC subgroup^31^. Using the DKFZ/Heidelberg CNS tumor classifier^25^, high correlation between these primary adult sellar SMARCB1/INI1-deficient tumors and ATRT-MYC was also observed. In contrast, using the sarcoma classifier^26^, no correlation between primary adult sellar SMARCB1/INI1-deficient tumors and sarcomas was observed, except for MRT. This suggests that primary adult sellar SMARCB1/INI1-deficient tumors had epigenetic profiles similar to the ATRT-MYC subgroup, but different from bona fide PES. Taken together, our findings indicate that the eight primary adult sellar SMARCB1/INI1-deficient tumors from this study should be better diagnosed as adult sellar ATRT rather than PES. In addition, DNA methylation profiling is recommended to differentiate the majority of (P)ES and (sellar) rhabdoid tumor.

In conclusion, primary adult sellar SMARCB1/INI1-deficient tumors are rare. Despite of the similarities in morphological, immunohistochemical and genetic characteristics with bona fide PES, primary adult sellar SMARCB1/INI1-deficient tumors showed similar epigenetic characteristics with pediatric ATRT-MYC subgroup, while clustered apart from bona fide PES. Thus, primary adult sellar SMARCB1/INI1-deficient tumors represent a subtype of ATRT with high CD34 expression. Accurate pathological diagnosis is crucial to guide the treatment after surgery. For instance, with a diagnosis of ATRT, high-dose chemotherapy and radiotherapy should be preferred^9,31^. While with a diagnosis of PES, extensive surgical resection and moderate adjuvant chemotherapy or radiotherapy are the main options to help prevent local relapse^18,36^.

## Supplementary information

Supplementary materials

## Data Availability

The data used and/or analyzed during the current study are available from the corresponding author upon reasonable request.
