# Supplementary material for: Primary adult sellar SMARCB1/INI1-deficient tumor represents a subtype of atypical teratoid/rhabdoid tumor
Source: Mod Pathol. 2022 Jul 8;35(12):1910–20. doi: 10.1038/s41379-022-01127-2 (PMC9708584; doi:10.1038/s41379-022-01127-2)
Supplement: Supplementary file 1 — Supplementary materials [file 41379_2022_1127_MOESM1_ESM.docx]

Supplementary Information for

**Primary adult sellar SMARCB1/INI1-deficient tumor represents a subtype of Atypical Teratoid/Rhabdoid Tumor**

Zejun Duan^1^*, Kun Yao^1^*, Shaomin Yang^2^, Yanming Qu^3^, Ming Ren^3^, Yongli Zhang^3^, Tao Fan^3^, Heqian Zhao^3^, Jie Gao^4^, Jing Feng^5^, Xiaolong Fan^5^, and Xueling Qi^1#^.

1. Department of Pathology, San Bo Brain Hospital, Capital Medical University.

2. Department of Pathology, School of Basic Medical Sciences, Third Hospital, Peking University Health Science Center.

3. Department of Neurosurgery, San Bo Brain Hospital, Capital Medical University.

4. Department of Medical Imaging, San Bo Brain Hospital, Capital Medical University

5. Beijing Key Laboratory of Gene Resource and Molecular Development, Laboratory of Neuroscience and Brain Development, School of Life Sciences, Beijing Normal University.

* These authors contributed equally to this work.

**Running title:** Primary adult sellar SMARCB1/INI1-deficient tumor

**# Corresponding author:**

Xueling Qi, M.D.

Department of Pathology, San Bo Brain Hospital, Capital Medical University, Beijing.

**This Word file includes:**

Supplementary Figs. 1 to 3

Supplementary Tables 1 to 3

**
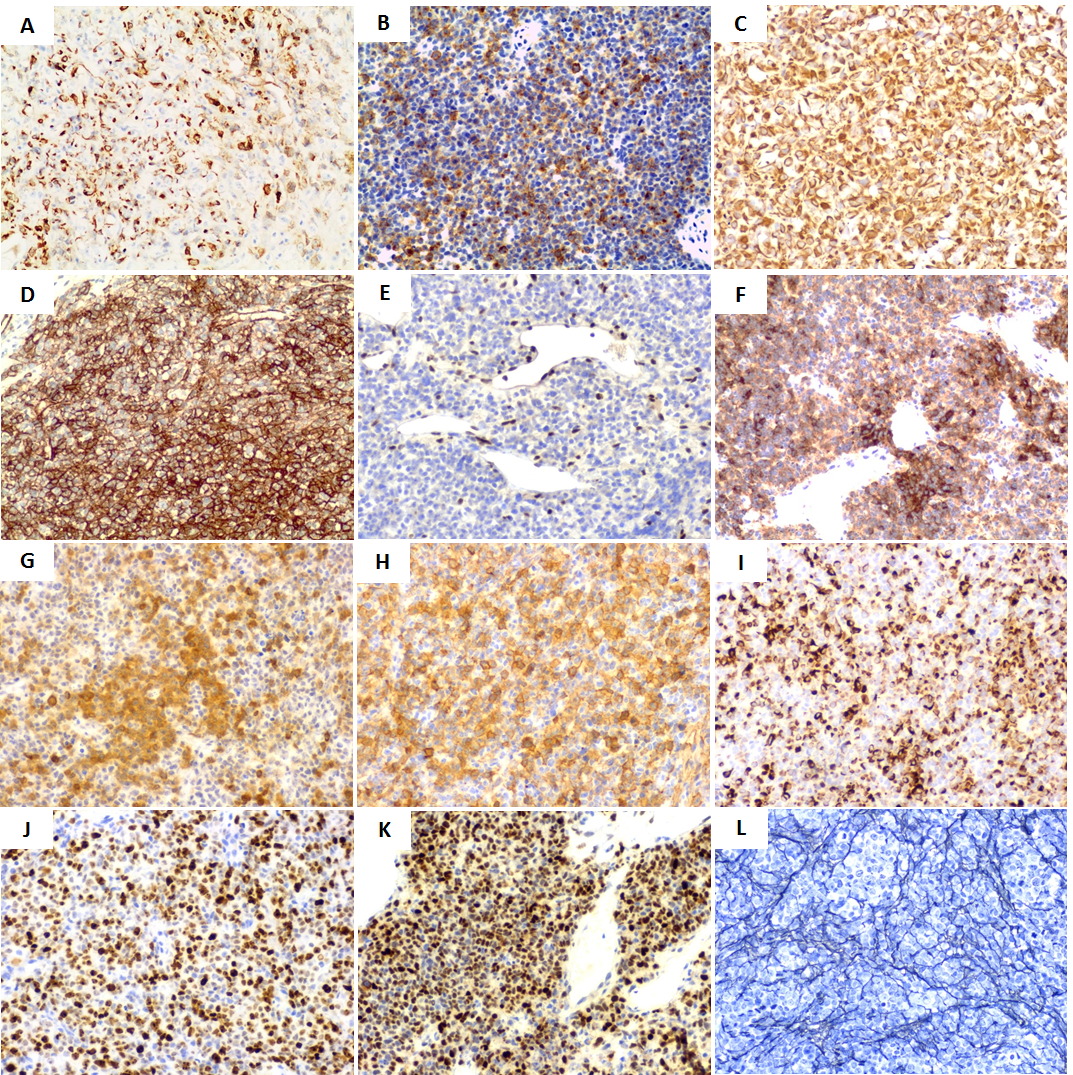
**

**Supplementary Fig. 1.** **Representative immunohistochemical features of the eight primary adult sellar SMARCB1/INI1-deficient tumors**. (A) Diffuse cytoplasmic AE1/AE3 staining in case # 3. (B) Cytoplasmic EMA staining in most tumor cells of case # 1. (C) Diffuse strong staining of Vimentin in case # 6. (D) Diffuse strong staining of CD34 in case # 3. (E) Lack of nuclear expression of INI1 protein in tumor cells of case # 7; note strong positive staining in endothelial cells. (F) Diffuse positive staining of Syn in case # 3. (G) Diffuse positive staining for MAP2 in case # 5. (H) Diffuse positive staining for SMA in case # 5. (I) Positive staining of Desmin in tumor cells of case # 6. (J) High MIB-1 (Ki-67) labeling in case # 5. (K) Strong TP53 expression in case # 3. (L) Abundant reticular fibers around tumor cells in case # 6. Images were taken at a magnification of 200x.


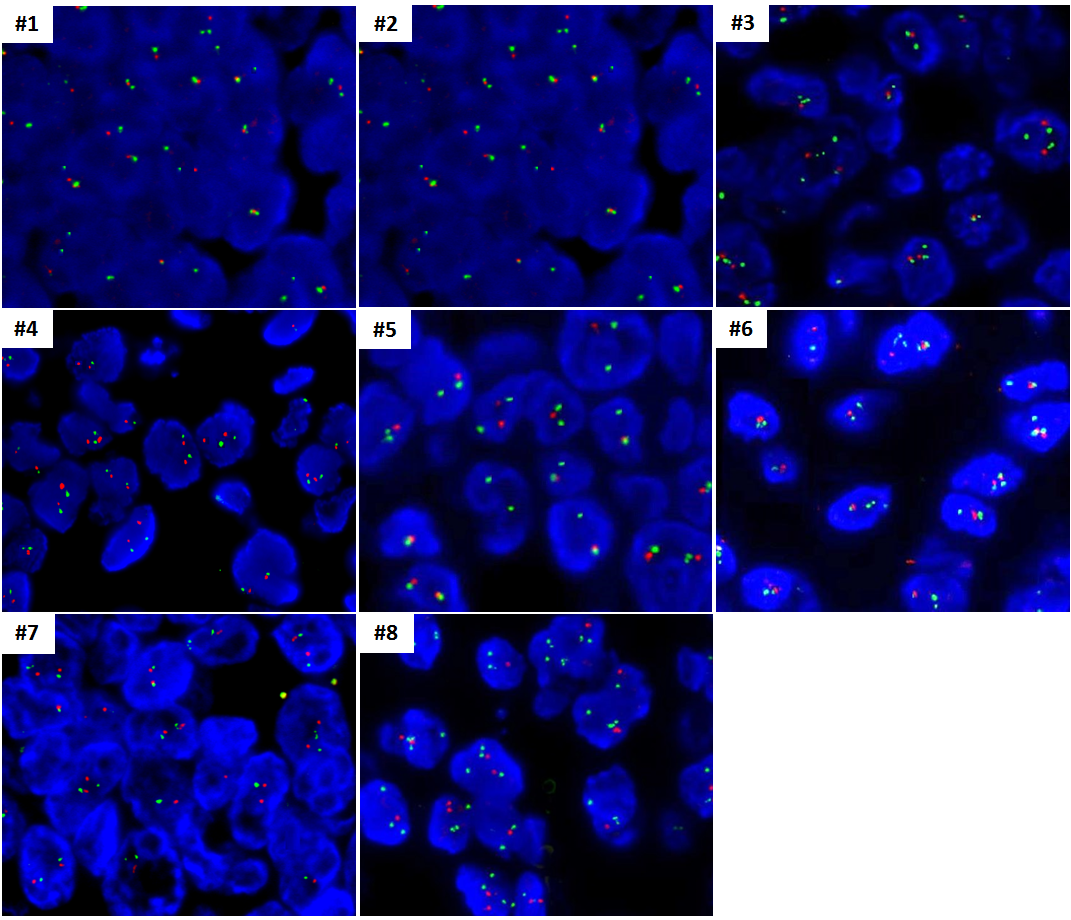


**Supplementary Fig. 2.** **Most primary adult sellar SMARCB1/INI1-deficient tumors harbored heterozygous deletion of *SMARCB1* locus.** Images shown are representative FISH results from cases # 1-8. Heterozygous deletion of *SMARCB1* with one hybridization signal in red for the *SMARCB1* locus, but two signals in green for the control *EWSR1* locus were found in all cases except cases # 4 and # 7.


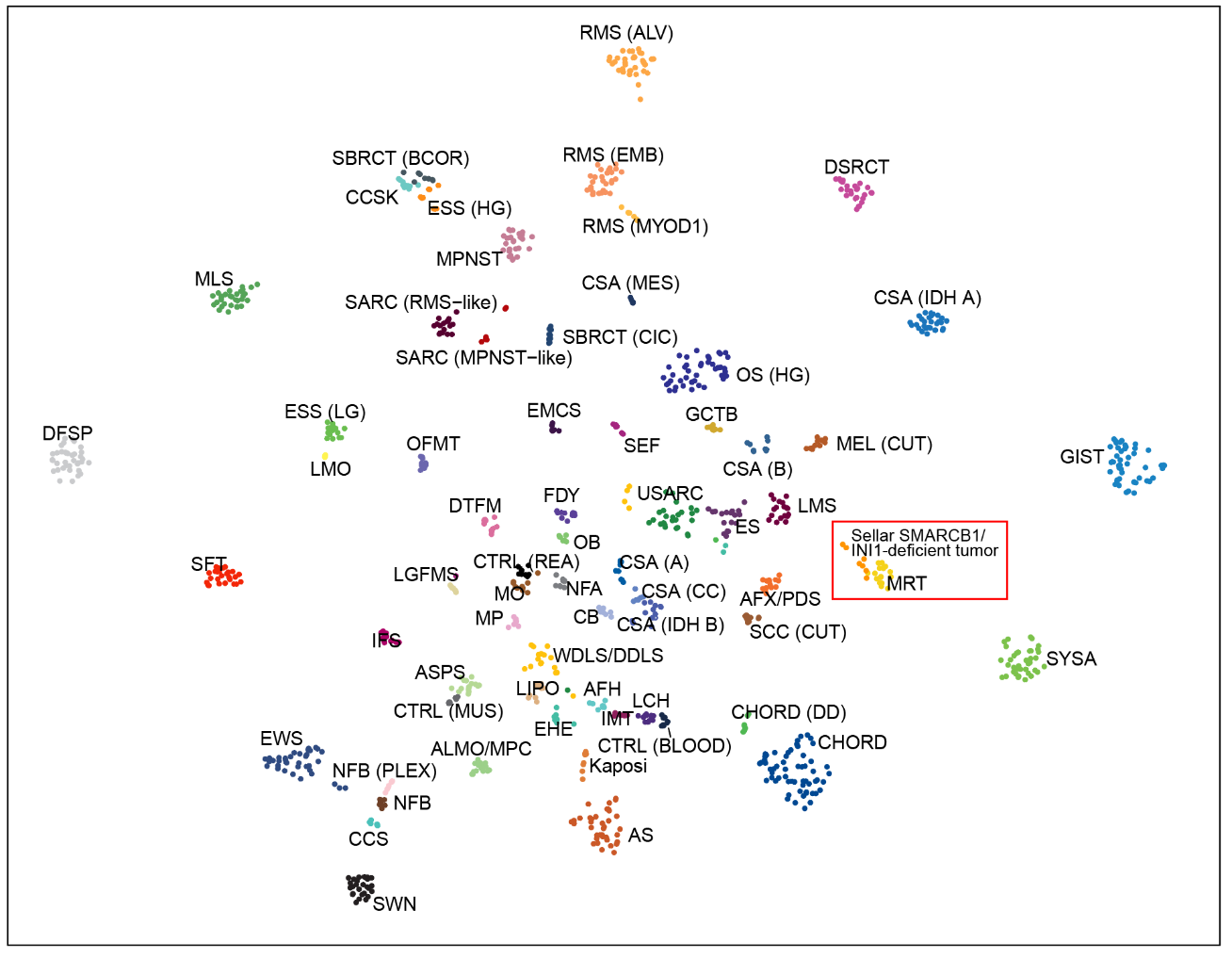


**Supplementary Fig. 3.** T-SNE cluster of DKFZ/Heidelberg sarcoma dataset^25^ and our primary adult sellar SMARCB1/INI1-deficient tumors based on DNA methylation data.

**Supplementary Table 1. 425-cancer-relevant genes**

| ABCB1(MDR1) | CDC73 | ERCC2 | IDH2 | MTOR | PRKACA | SOX14 |
| --- | --- | --- | --- | --- | --- | --- |
| ABCB4 | CDH1 | ERCC3 | IFNG | MUTYH | PRKACG | SOX2 |
| ABCC2(MRP2) | CDK10 | ERCC4 | IFNGR1 | MYC | PRKAR1A | SOX21 |
| ADH1A | CDK12 | ERCC5 | IGF1R | MYCL | PRKCI | SPOP |
| ADH1B | CDK4 | ESR1 | IGF2 | MYCN | PRKDC | SPRY4 |
| ADH1C | CDK6 | ETV1 | IKBKE | MYD88 | PRSS1 | SRC |
| AIP | CDK8 | ETV4 | IKZF1 | MYH9 | PRSS3 | SRY |
| AKT1 | CDKN1A | ETV6 | IL7R | NAT1 | PTCH1 | STAG2 |
| AKT2 | CDKN1B | EWSR1 | INPP4B | NBN | PTEN | STAT3 |
| AKT3 | CDKN1C | EXT1 | IRF2 | NCOR1 | PTK2 | STK11 |
| ALDH2 | CDKN2A | EXT2 | JAK1 | NF1 | PTPN11 | STMN1 |
| ALK | CDKN2B | EZH2 | JAK2 | NF2 | PTPN13 | STT3A |
| AMER1 | CDKN2C | FANCA | JAK3 | NFE2L2 | PTPRD | SUFU |
| APC | CEBPA | FANCC | JARID2 | NFKBIA | QKI | TAP1 |
| AR | CEP57 | FANCD2 | JUN | NKX2-1 | RAC1 | TAP2 |
| ARAF | CHD4 | FANCE | KDM5A | NKX2-4 | RAC3 | TEK |
| ARID1A | CHEK1 | FANCF | KDM6A | NOTCH1 | RAD50 | TEKT4 |
| ARID1B | CHEK2 | FANCG | KDR(VEGFR2) | NOTCH2 | RAD51 | TERC |
| ARID2 | CREBBP | FANCI | KEAP1 | NOTCH3 | RAD51B | TERT |
| ARID5B | CRKL | FANCL | KIF1B | NPM1 | RAD51C | TET2 |
| ASCL4 | CSF1R | FANCM | KIF5B | NQO1 | RAD51D | TGFBR2 |
| ASXL1 | CTCF | FAT1 | KIT | NRAS | RAD54L | THADA |
| ATF1 | CTLA4 | FBXW7 | KITLG | NRG1 | RAF1 | TMEM127 |
| ATIC | CTNNB1 | FGF19 | KLLN | NSD1 | RARA | TMPRSS2 |
| ATM | CUL3 | FGFR1 | KMT2A(MLL) | NTRK1 | RARG | TNFAIP3 |
| ATR | CUX1 | FGFR2 | KMT2B | NTRK2 | RASGEF1A | TNFRSF11A |
| ATRX | CXCR4 | FGFR3 | KMT2C | NTRK3 | RB1 | TNFRSF14 |
| AURKA | CYLD | FGFR4 | KMT2D(MLL2) | PAK3 | RECQL4 | TNFRSF19 |
| AURKB | CYP19A1 | FH | KRAS | PALB2 | RELN | TNFSF11 |
| AXIN2 | CYP2A13 | FLCN | LHCGR | PALLD | RET | TOP1 |
| AXL | CYP2A6 | FLT1(VEGFR1) | LMO1 | PARK2 | RHOA | TOP2A |
| B2M | CYP2A7 | FLT3 | LRP1B | PARP1 | RICTOR | TP53 |
| BAD | CYP2B6*6 | FLT4 | LYN | PARP2 | RNF43 | TP63 |
| BAI3 | CYP2C19*2 | FOXA1 | LZTR1 | PAX5 | ROS1 | TPMT |
| BAK1 | CYP2C9*3 | FOXP1 | MAP2K1(MEK1) | PBRM1 | RPTOR | TSC1 |
| BAP1 | CYP2D6 | FRG1 | MAP2K2(MEK2) | PDCD1(PD1) | RRM1 | TSC2 |
| BARD1 | CYP3A4*4 | GATA1 | MAP2K4 | PDCD1LG2(PD-L2) | RUNX1 | TSHR |
| BAX | CYP3A5 | GATA2 | MAP3K1 | PDE11A | RUNX1T1 | TTF1 |
| BCL2 | DAXX | GATA3 | MAP3K4 | PDGFRA | SBDS | TUBB3 |
| BCL2L11(BIM) | DDR2 | GATA4 | MAP4K3 | PDGFRB | SDC4 | TUBB4A |
| BCR | DENND1A | GATA6 | MAX | PDK1 | SDHA | TUBB4B |
| BIRC3 | DHFR | GNA11 | MCL1 | PGR | SDHB | TUBB6 |
| BLM | DICER1 | GNAQ | MDM2 | PHOX2B | SDHC | TYMS |
| BMPR1A | DLL3 | GNAS | MDM4 | PIK3C3 | SDHD | U2AF1 |
| BRAF | DNMT3A | GRIN2A | MECOM | PIK3CA | 9-Sep | UGT1A1 |
| BRCA1 | DPYD | GRM3 | MED12 | PIK3R1 | SETBP1 | VAMP2 |
| BRCA2 | DUSP2 | GRM8 | MEF2B | PIK3R2 | SETD2 | VEGFA |
| BRD4 | EGFR | GSTM1 | MEN1 | PKHD1 | SF3B1 | VHL |
| BRIP1 | EML4 | GSTM4 | MET | PLAG1 | SGK1 | WAS |
| BTG2 | EP300 | GSTM5 | MGMT | PLK1 | SLC34A2 | WISP3 |
| BTK | EPAS1 | GSTP1 | MITF | PMS1 | SLC3A2 | WRN |
| BUB1B | EPCAM | GSTT1 | MLH1 | PMS2 | SLC7A8 | WT1 |
| c11orf30 | EPHA2 | HDAC2 | MLH3 | POLD1 | SMAD2 | XPA |
| CASP8 | EPHA3 | HDAC9 | MLLT1 | POLD3 | SMAD3 | XPC |
| CBL | EPHA5 | HGF | MLLT3 | POLE | SMAD4 | XRCC1 |
| CBLB | EPHB2 | HLA-A | MLLT4 | POLH | SMAD7 | YAP1 |
| CCND1 | ERBB2(HER2) | HNF1A | MPL | POT1 | SMARCA4 | ZNF2 |
| CCNE1 | ERBB2IP | HNF1B | MRE11A | PPARD | SMARCB1 | ZNF217 |
| CD274(PD-L1) | ERBB3 | HRAS | MSH2 | PPP2R1A | SMO | ZNF703 |
| CD74 | ERBB4 | HSD3B1 | MSH6 | PRDM1 | SOS1 | CDA |
| ERCC1 | IDH1 | MTHFR | PRF1 | SOX1 |  |  |

**Supplementary Table 2. Clinical characteristics of three SMARCB1/INI1-deficient tumor types for comparative analysis**

|  | **Bona fide PES** | **ATRT** | | **PDC** |
| --- | --- | --- | --- | --- |
| **Number of cases** | 6 | 14 | | 5 |
| **Age** | Adult (range: 20-59y, mean: 32y) | 9 children (range: 10-39ms, mean: 20.6ms) | 2 teenagers and 3 adults (range: 15-46y, mean: 25y) | children (range: 3-16y, mean: 6.4y) |
| **Sex (F: M)** | 2:4 | 4:10 | | 3:2 |
| **Localization** | 3 in limbs,2 genital organ, and 1 in cervical spine | 8 in supratentorial region (2 cerebral hemispheres, 2 lateral ventricle, 2 third ventricle, 1 pineal region, and 1hypothalamus); 6 in infratentorial region (5 cerebellar hemisphere and the fourth ventricle, and 1 cerebellopontine angle) | | 5 in clivus with or without involvement of sellar region and upper cervical spine |
| **Outcome** | NA | 5 patients deceased, OS (range:2 weeks to 8 ms); 3 patients alive, follow-up time (range: 8-46ms) | | All patients deceased, OS (range: 2-17ms, mean: 5.4ms) |

NA: not available

**Supplementary Table 3．Differential expression patterns of key IHC markers in the four SMARCB1/INI1-deficient tumor types examined**

|  | **Primary adult sellar SMARCB1/INI1-deficient tumors** | **Bona fide PES** | **ATRT** | **PDC** |  |
| --- | --- | --- | --- | --- | --- |
| **CD34** | 75% (6/8) | 100% (6/6) | 21.4% (3/14) ^a^ | 20%(1/5)^b^ |  |
| **ERG** | 0 (0/8) | 50% (3/6) | 14.3% (2/14) | 0 (0/5) |  |
| **FLI-1** | 100% (8/8) | 80% (4/5) | 92.9% (13/14) | 100% (5/5) |  |
| **SALL4** | 37.5% (3/8) | 16.67% (1/6) | 50% (7/14) | 0 (0/5) |  |
| **β-catenin*** | 100% (5/5) | 80% (4/5) | 92.9% (13/14) | 100% (5/5) |  |
| **Brachyury** | 0 (0/8) | 0 (0/6) | 0 (0/14) | 100% (5/5) |  |
| ^a^ CD34 positive ATRTs included 2 children and 1 adult, about 5% and 20% of tumor cells expressed CD34 respectively in children cases, and about 5% in adult case. ^b^ CD34 was positive staining in about 20% of tumor cells.* β-catenin stains positively on the cell membrane and occasionally also in the cytoplasm. | | | | | |
